# When Hormones Shape the Mind: Neuropsychiatric Manifestations in a Patient With Congenital Adrenal Hyperplasia and Genital Ambiguity

**DOI:** 10.7759/cureus.97582

**Published:** 2025-11-23

**Authors:** David Galindo, Carolina Gonzalez, Daniela Osorno, Laura Carolina Baños Pabon

**Affiliations:** 1 Psychiatry and Behavioral Sciences, Hospital Mental de Antioquia, Bello, COL; 2 Psychiatry, Hospital Mental de Antioquia, Bello, COL; 3 Psychiatry Program, Corporación Universitaria Uniremington, Medellin, COL; 4 Psychiatry Program, Corporacion Universitaria Uniremington, Medellin, COL

**Keywords:** 21-hydroxylase deficiency, bipolar affective disorder, congenital adrenal hyperplaisa, genital ambiguity, neuropsychiatric manifestations

## Abstract

Congenital adrenal hyperplasia (CAH) due to 21-hydroxylase deficiency can produce neuropsychiatric manifestations through cortisol deficiency, adrenocorticotropic hormone (ACTH)-driven hyperandrogenism, and the cumulative effects of long-term glucocorticoid therapy. We report a 33-year-old transgender male (sex assigned female at birth) with a history of neonatal genital ambiguity surgically corrected and lifelong prednisolone replacement who presented with a five-day course of irritability, expansive mood, decreased need for sleep, erratic behavior, heteroaggression, and psychotic symptoms. Physical examination showed Cushingoid stigmata (moon facies, dorsocervical fat pad, centripetal adiposity, violaceous striae, and acral edema). Initial management included continuation of divalproex and clozapine; lithium was discontinued due to renal dysfunction and clinical worsening. Endocrine evaluation demonstrated low morning cortisol with elevated ACTH, markedly increased total and free testosterone with low-normal gonadotropins, and severe hypothyroidism, findings consistent with primary adrenal insufficiency and adrenal-origin hyperandrogenism in the setting of CAH. Given persistent manic psychosis and limited pharmacologic options, modified electroconvulsive therapy (ECT) under anesthesia and muscle relaxation was initiated (12 bilateral sessions) without complications, with progressive stabilization of mood, attenuation of psychotic content, and normalization of sleep-wake cycles. The patient was discharged in improved condition with endocrine and psychiatric follow-up, including thyroid replacement and reassessment of glucocorticoid strategy. This case underscores the diagnostic interplay between endocrine dysregulation and affective pathology, the importance of multidisciplinary coordination (psychiatry, endocrinology, and clinical genetics), and the therapeutic value of transfer energy capacitive and resistive (TECAR) when conventional mood-stabilizing regimens are constrained by medical comorbidity. Early recognition and targeted hormonal optimization may reduce iatrogenic risk and improve neuropsychiatric outcomes in adults with CAH.

## Introduction

Congenital adrenal hyperplasia (CAH) due to CYP21A2 mutations is classified into two major forms: classic CAH, encompassing the salt-wasting and simple-virilizing phenotypes with early-onset cortisol deficiency and androgen excess, and nonclassic CAH, a milder variant with partial 21-hydroxylase activity that typically presents later with hyperandrogenic symptoms. Most cases of CAH result from CYP21A2 mutations affecting the 21-hydroxylase enzyme, accounting for over 90% of presentations. Classic CAH occurs in approximately one in 15,000 live births, while the nonclassic form is more common, with a prevalence of about one in 1,000 individuals [1]. This enzyme catalyzes the conversion of 17-hydroxyprogesterone to 11-deoxycortisol and progesterone to 11-deoxycorticosterone [2]. Its deficiency results in impaired cortisol and aldosterone synthesis, compensatory adrenocorticotropic hormone (ACTH) hypersecretion, and excessive adrenal androgen production [3]. The ensuing hormonal disequilibrium produces variable clinical phenotypes ranging from salt-wasting crises to virilization and genital ambiguity in 46,XX individuals, and in rare cases, undervirilization in 46,XY individuals [4].

Lifelong glucocorticoid replacement, typically with hydrocortisone, remains the cornerstone of therapy, aiming to suppress ACTH overproduction and normalize androgen levels [5]. However, replicating the physiological circadian rhythm of cortisol secretion with standard hydrocortisone formulations remains challenging, frequently resulting in cyclical periods of both overexposure and underexposure [6]. Chronic supraphysiological doses can induce iatrogenic Cushingoid states, while suboptimal replacement may lead to persistent androgen excess, each contributing to neuroendocrine and behavioral dysregulation [7]. A flattened cortisol rhythm has been linked to cognitive and affective disturbances, particularly during developmental stages.

Glucocorticoid receptors are widely distributed in brain regions critical for emotion and stress regulation, including the prefrontal cortex, hippocampus, amygdala, and cerebellum [8]. Functional neuroimaging studies have demonstrated alterations in white matter integrity, hippocampal volume, and amygdala reactivity in individuals with CAH, supporting a neurobiological substrate for mood and behavioral changes [9]. Moreover, the interplay between cortisol, dehydroepiandrosterone (DHEA), and pregnenolone - neurosteroids capable of modulating GABAergic and glutamatergic neurotransmission - contributes to emotional instability and cognitive variability.

Exogenous glucocorticoids themselves are known to precipitate neuropsychiatric adverse effects, including anxiety, mania, and psychosis, in approximately 10% of exposed individuals [10]. These findings underscore the bidirectional and complex relationship between endocrine regulation and psychiatric symptomatology [11].

We present the case of a transgender male with CAH and genital ambiguity who developed an acute manic episode consistent with bipolar affective disorder. This case highlights the intricate neuroendocrine mechanisms linking steroidogenic dysfunction and affective regulation, emphasizing the need for coordinated endocrine-psychiatric management in patients with disorders of sex development.

## Case presentation

A 33-year-old individual registered as female but identifying as a transgender male from El Peñol, Antioquia, Colombia, was admitted to the Hospital Mental de Antioquia (HOMO) on September 17, 2025, accompanied by his sister, after a five-day history of behavioral and affective instability. He was single, unemployed, and occasionally worked informally, collecting and selling scrap metal. His past medical history included bipolar I disorder, mild intellectual disability, primary hypothyroidism, and obesity. At birth, he was diagnosed with ambiguous genitalia and underwent corrective surgery during the neonatal period. Since that time, he had been receiving long-term prednisolone as hormonal replacement for CAH. His sister had a confirmed diagnosis of CAH with overlapping clinical features, while the family history of bipolar affective disorder was traced to his father. Neither parent exhibited phenotypic features of CAH, and no further genetic testing had been conducted within the family.

In the days preceding admission, relatives reported increasing irritability, expansive mood, reduced need for sleep, and excessive energy, accompanied by erratic extramural wandering, impulsive borrowing of money, and episodes of heteroaggression involving destruction of windows and doors. He expressed grandiose delusions (“I have a business empire,” “I can perform miracles”) and mystical religious delusions, along with auditory command hallucinations. These symptoms led to hospital admission with a diagnosis of bipolar affective disorder, current manic episode with psychotic features.

**Figure 1:**
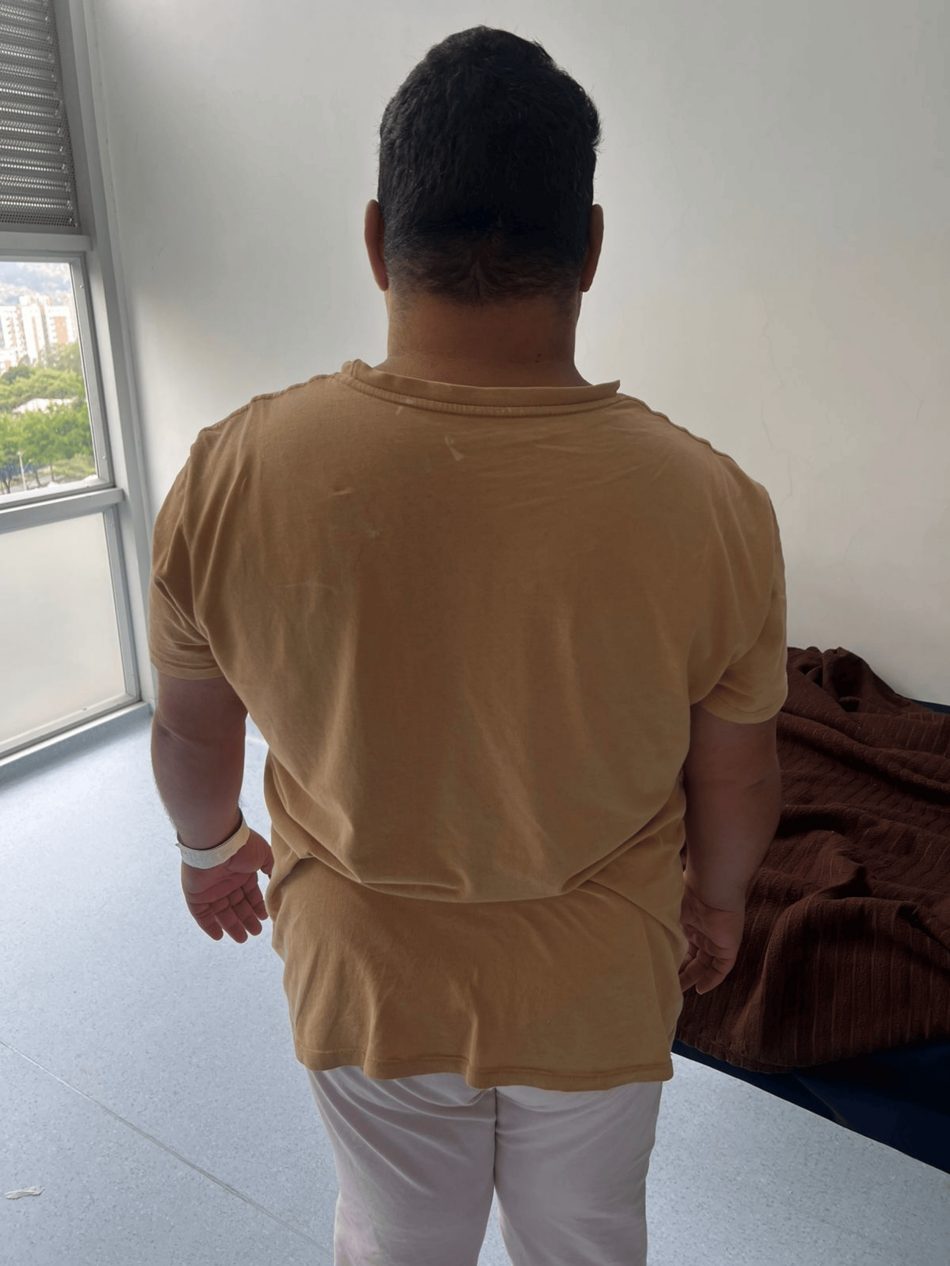
Dorsal view of the patient showing features of iatrogenic Cushing’s syndrome. Central obesity with a prominent dorsocervical fat pad (“buffalo hump”) and rounded body contour, consistent with adipose redistribution secondary to chronic corticosteroid therapy in a patient with congenital adrenal hyperplasia. The patient during hospitalization exhibited physical characteristics of iatrogenic Cushing’s syndrome secondary to prolonged prednisolone therapy. Note the rounded facies, supraclavicular fat accumulation, central adiposity, and violaceous striae on the abdomen and flanks.

**Figure 2:**
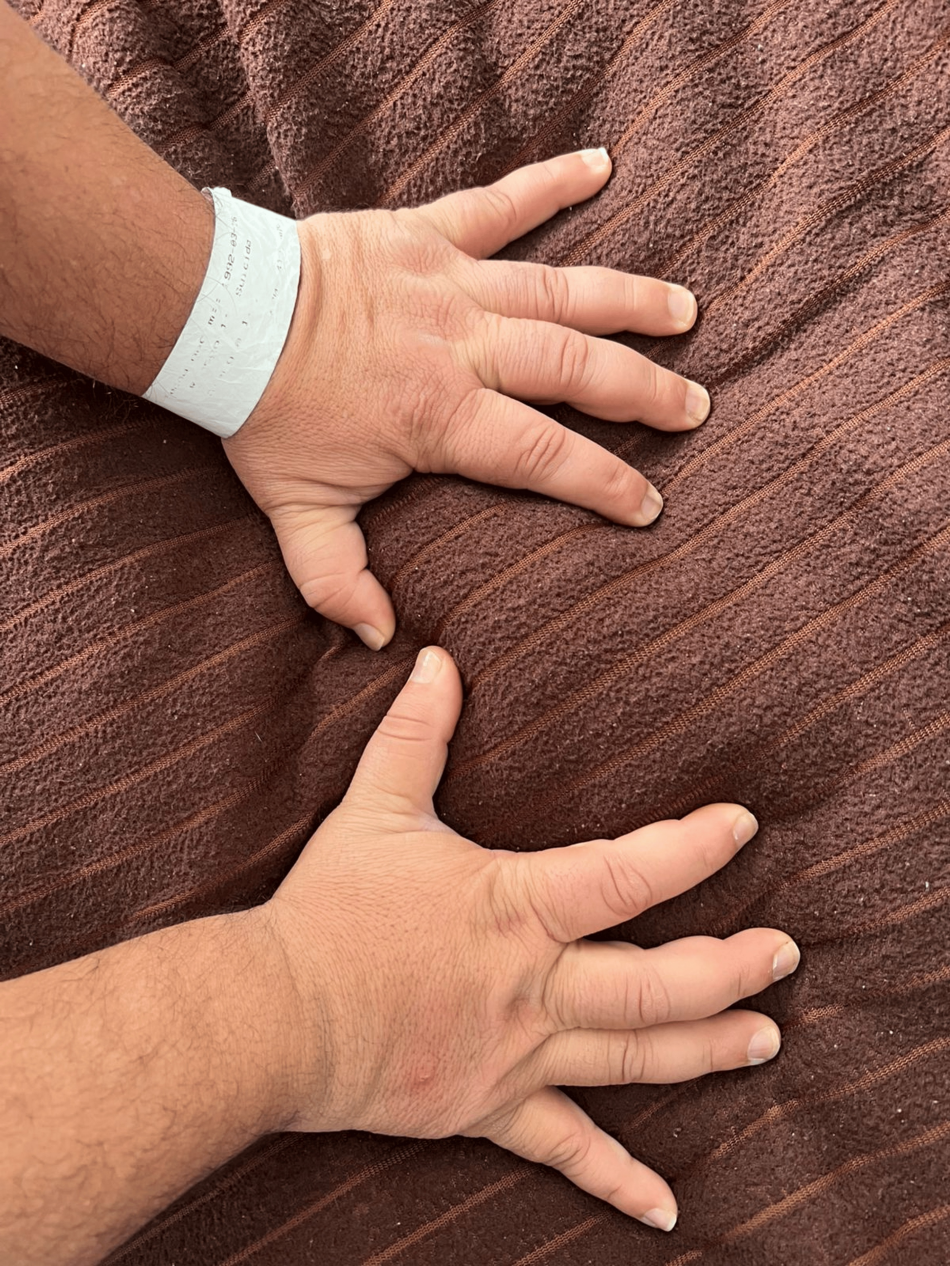
Hands of the patient showing physical signs of iatrogenic Cushing’s syndrome. Bilateral swelling and thickened digits with loss of joint definition, consistent with soft tissue edema and redistribution of adipose tissue due to chronic corticosteroid exposure in a patient with congenital adrenal hyperplasia treated with long-term prednisolone.

On admission, the patient was alert, oriented, and cooperative but exhibited pressured speech, euphoric mood, and psychomotor agitation. Physical examination revealed stigmata of chronic corticosteroid exposure, including facial rounding (“moon face”), dorsocervical fat pad (“buffalo hump”), central obesity with thin extremities, violaceous abdominal striae, mild hirsutism, and acneiform eruptions, consistent with iatrogenic Cushingoid features (Figure 1). The hands of the patient showed physical signs of iatrogenic Cushing’s syndrome (Figure 2). Vital signs were stable, and there were no focal neurological deficits.

The patient was receiving outpatient treatment with sodium divalproate, clozapine, and lithium carbonate. Lithium was discontinued shortly after admission due to the appearance of renal dysfunction and exacerbation of manic symptoms. Serial laboratory evaluations revealed mild renal impairment and severe hypothyroidism, prompting reintroduction of levothyroxine. During the second week of hospitalization, given the background of genital ambiguity, obesity, and chronic corticosteroid use, endocrinological testing was ordered to assess hormonal function and its possible contribution to mood instability. The hormonal profile demonstrated decreased cortisol with elevated ACTH, consistent with primary adrenal insufficiency secondary to 21-hydroxylase deficiency. Testosterone levels were elevated, whereas luteinizing hormone (LH) and follicle-stimulating hormone (FSH) were suppressed, indicating adrenal hyperandrogenism typical of CAH. The coexistence of enzyme deficiency and long-term corticosteroid exposure produced a dual mechanism of hypothalamic-pituitary-adrenal (HPA) axis suppression and neurosteroid imbalance, both contributing to affective dysregulation and manic exacerbation.

Table 1 shows a summary of the laboratory and endocrinological data obtained during hospitalization. The results demonstrate reduced cortisol, elevated ACTH, increased testosterone, and suppressed gonadotropins, compatible with primary adrenal insufficiency due to 21-hydroxylase deficiency and adrenal hyperandrogenism. Mild renal dysfunction and hypothyroidism were also identified and corrected during treatment.

**Table 1:** Summary of serial laboratory parameters during hospitalization with reference ranges. Laboratory findings revealed severe hypothyroidism with markedly elevated thyroid-stimulating hormone (TSH) and biochemical evidence of primary adrenal insufficiency, indicated by low morning cortisol and elevated adrenocorticotropic hormone (ACTH). The androgen profile showed increased total and free testosterone with low-normal luteinizing hormone (LH) and follicle-stimulating hormone (FSH), consistent with adrenal hyperandrogenism secondary to congenital adrenal hyperplasia. Mild transient renal dysfunction was noted, with initial creatinine elevation that later normalized. Lithium levels remained subtherapeutic during hospitalization. Reference ranges are included for interpretation.

Parameter	Reference range	Day 1	Day 3	Day 7	Day 9	Day 15
Creatinine	0.6–1.2 mg/dL	1.33	1.13	1.25	1.3	
Lithium level	0.8–1.2 mEq/L	0.650		0.5		
TSH	0.4–4.5 mIU/L	87.83				
Valproic acid	50–125 µg/mL	94				
Glycemia	70–99 mg/dL	103				
HbA1C	<5.7%		4.7%			
Vitamin B12	200–900 pg/mL				481.66	
Urea nitrogen (BUN)	7–20 mg/dL				15.7	
Urea	15–40 mg/dL				33.6	
Total testosterone	13.84–53.35 ng/dL					215.65 ng/dL
Free testosterone	0.1–6.3 pg/mL					25.31 pg/mL
ACTH	7.2–63.3 pg/mL					64.10 pg/mL
Cortisol a.m.	3.7–19.4 ug/dL					2.50 ug/dL
Luteinizing hormone	Follicular phase: 1.8–11.78 mUI/mL					1.60 mUI/mL
Follicle-stimulating hormone	Follicular phase: 3.03–8.08 mUI/mL					4.03 mUI/mL
Estradiol	Follicular phase: 21–251 pg/mL					45.42 pg/mL

Pharmacological management was optimized with therapeutic doses of valproate and gradual titration of clozapine; however, the patient continued to display psychomotor agitation, expansiveness, and residual delusional content. Because of limited pharmacological response and contraindication to lithium, modified electroconvulsive therapy (ECT) under anesthesia and muscle relaxation was initiated. Twelve bilateral sessions were administered over three weeks without complications, resulting in progressive improvement. The patient’s affect stabilized, psychotic symptoms diminished, and sleep-wake rhythm normalized. The therapeutic effect was attributed to the neurobiological actions of ECT, including modulation of monoaminergic transmission, enhancement of cortical excitability, and reactivation of HPA axis regulation.

Throughout hospitalization, the patient’s behavior evolved from pronounced irritability and disorganization to calmness and emotional control. By the fourth week, he verbalized insight into his previous behaviors, expressed embarrassment, and described feeling “calm and clear-minded.” Nursing notes confirmed adequate adherence to treatment, stable circadian rhythms, and proper social interaction. After multidisciplinary evaluation by psychiatry, endocrinology, and clinical genetics, the diagnosis of CAH with adrenal insufficiency was confirmed. The patient was discharged in stable condition following marked remission of manic and psychotic symptoms, corticosteroid replacement therapy, along with psychiatric follow-up. His clinical improvement illustrates the intricate interaction between steroidogenic dysfunction, chronic glucocorticoid exposure, and affective regulation, as well as the therapeutic role of ECT in complex neuroendocrine mood disorders.

## Discussion

CAH due to 21-hydroxylase deficiency is the most common form of adrenal steroidogenic dysfunction, accounting for over 90% of cases [12]. This autosomal recessive disorder leads to impaired synthesis of cortisol and aldosterone, compensatory ACTH hypersecretion, and excessive adrenal androgen production. Although primarily endocrinological, CAH frequently exhibits neuropsychiatric manifestations arising from chronic hormonal disequilibrium, disrupted HPA regulation, and the cumulative effects of long-term glucocorticoid therapy [13].

In the present case, the patient - a transgender male with a history of ambiguous genitalia corrected in infancy - had been treated since birth with prednisolone as hormonal replacement. Prolonged exposure to supraphysiologic doses of synthetic corticosteroids produced iatrogenic Cushingoid stigmata (moon facies, dorsocervical fat pad, central obesity, and violaceous striae), accompanied by affective and behavioral dysregulation. Chronic corticosteroid therapy is known to alter neuroendocrine feedback mechanisms and increase susceptibility to mood disorders, including mania and psychosis, in approximately 10% of exposed individuals. These neuropsychiatric effects result from glucocorticoid-mediated dysregulation of limbic and prefrontal cortical circuits, impaired neuroplasticity, and altered monoaminergic transmission [14].

The clinical presentation - irritability, expansive mood, decreased need for sleep, psychomotor acceleration, and psychotic symptoms - was consistent with a steroid-induced manic episode superimposed on an underlying bipolar disorder. Biochemical testing confirmed low morning cortisol with elevated ACTH, suggesting primary adrenal insufficiency, and markedly increased total and free testosterone with suppressed gonadotropins, consistent with adrenal hyperandrogenism secondary to CAH. The simultaneous presence of cortisol deficiency and androgen excess creates a neurobiological environment characterized by HPA axis instability and neurosteroid imbalance, both contributing to affective dysregulation.

Functional neuroimaging in CAH has demonstrated white-matter abnormalities and structural changes in the amygdala and hippocampus, regions crucial for emotional regulation [15]. Moreover, alterations in neurosteroids such as DHEA and pregnenolone-potent GABAergic and glutamatergic modulators can exacerbate anxiety, irritability, and impulsivity. In this context, the long-term use of prednisolone rather than hydrocortisone likely intensified the hormonal disequilibrium. Hydrocortisone remains the preferred replacement therapy for CAH, as it more closely mimics the physiological circadian rhythm of cortisol, reducing the risk of iatrogenic Cushing’s syndrome and neuropsychiatric complications.

Management in such complex scenarios requires a multidisciplinary approach that integrates psychiatry, endocrinology, internal medicine, and clinical genetics. Collaborative monitoring of adrenal, gonadal, and thyroid function is essential to prevent treatment-related metabolic and neuropsychiatric consequences. Endocrine follow-up should focus on optimizing glucocorticoid regimens, minimizing cumulative steroid exposure, and addressing comorbid autoimmune hypothyroidism or metabolic syndrome. Psychiatric intervention should consider the dynamic interaction between endocrine dysfunction and psychotropic drug metabolism, especially in the context of renal or hepatic compromise.

Given the limited response to pharmacological treatment and contraindication to lithium, modified ECT was introduced, producing progressive improvement in affective and psychotic symptoms. Beyond its recognized efficacy in severe and treatment-resistant mood episodes, ECT exerts neuroendocrine effects that may restore HPA axis balance, enhance monoaminergic regulation, and modulate stress-related neurocircuitry [16]. In patients with endocrine-psychiatric comorbidities, ECT represents a safe and effective therapeutic strategy when pharmacologic interventions are insufficient or contraindicated.

From a broader perspective, this case underscores the need to conceptualize CAH not merely as an endocrine disorder but as a neuroendocrine condition with systemic and psychiatric dimensions. Multidisciplinary coordination facilitates early detection of hormonal imbalances that exacerbate mood instability, allowing timely adjustments in both endocrine and psychiatric therapy. Moreover, structured integration of ECT within such treatment frameworks can provide a neurobiologically targeted intervention, complementing hormonal optimization and improving long-term outcomes [17].

In conclusion, this case exemplifies the intricate intersection between hormonal dysregulation and affective pathology in CAH. The coexistence of congenital enzyme deficiency, chronic corticosteroid exposure, and affective vulnerability resulted in a severe manic episode responsive to comprehensive, interdisciplinary management. Optimizing glucocorticoid therapy, ensuring continuous endocrine follow-up, and incorporating ECT as part of a multimodal treatment strategy are crucial for achieving sustained psychiatric and metabolic stabilization in these complex neuroendocrine disorders.

## Conclusions

This case illustrates the intricate interaction between congenital adrenal hyperplasia, chronic corticosteroid exposure, and mood instability. The overlap of endocrine dysfunction and psychiatric symptoms highlights the need for multidisciplinary evaluation and tailored hormonal management. The patient’s improvement following ECT underscores its value as a therapeutic option in severe, treatment-resistant affective episodes within neuroendocrine disorders.
